# Application of transesophageal echocardiography combined with FloTrac monitoring in cardiac valve replacement surgery

**DOI:** 10.3389/fcvm.2025.1667017

**Published:** 2025-10-31

**Authors:** Yunpeng Li, Ying Liu, Dandan Zhang

**Affiliations:** ^1^ Department of Ultrasound, Dongyang People’s Hospital Affiliated to Wenzhou Medical University, Jinhua, China; ^2^ Department of Anesthesiology, Dongyang People’s Hospital Affiliated to Wenzhou Medical University, Jinhua, China

**Keywords:** transesophageal echocardiography, FloTrac, cardiac valve replacement surgery, postoperative cognitive dysfunction, hemodynamic monitoring

## Abstract

**Objective:**

To compare intraoperative hemodynamics between transesophageal echocardiography (TEE) combined with FloTrac vs. TEE with invasive arterial pressure monitoring, and to examine associations with postoperative cognitive dysfunction (POCD) in patients undergoing cardiac valve replacement.

**Methods:**

A retrospective matched-cohort study included 162 patients (81 per group) matched by surgical type, ASA classification, age, and cardiopulmonary bypass time. Hemodynamic parameters were measured at four time points (T1–T4). Linear mixed-effects models assessed group, time, and interaction effects. Exploratory logistic regression preserving the matched design evaluated associations with POCD.

**Results:**

Group effects were significant for heart rate (HR, *F* = 6.79, *p* = 0.009), cardiac output (CO, *F* = 17.05, *p* < 0.001), cardiac index (CI, *F* = 16.49, *p* < 0.001), and stroke volume variation (SVV, *F* = 18.73, *p* < 0.001). Group × time interactions were observed for MAP, CVP, HR, SV, CI, SVRI, SVV, VTI, and LVEDV (all *p* < 0.05). Pearson correlations at T3 were weak (SV vs. CI *r* = 0.274; FAC vs. SVRI *r* = −0.220). Postoperative complication rates, including POCD (9.9% vs. 18.5%, OR = 0.48, 95% CI: 0.19–1.21, *p* = 0.115), were not significantly different. HR at T2 and SVRI at T4 showed nominal associations with POCD, but predictive ability was limited.

**Conclusion:**

TEE combined with FloTrac provides a more detailed intraoperative hemodynamic assessment and reveals distinct temporal trends compared to invasive arterial pressure monitoring. These differences did not correspond to changes in clinical outcomes in this cohort, but the observations may inform the design of future studies on hemodynamic monitoring strategies and POCD risk.

## 1 Introduction

Cardiac valve replacement is a high-risk surgical procedure involving the repair or replacement of cardiac structures and is often accompanied by significant perioperative challenges. Maintaining stable hemodynamics throughout the operation is crucial, as instability has been linked to adverse outcomes (1). Hemodynamic monitoring plays a vital role in cardiac surgery (2). Conventional techniques such as invasive arterial blood pressure monitoring and central venous pressure (CVP) monitoring provide essential baseline parameters but fall short in capturing the dynamic functional changes of the heart in real time.

Transesophageal echocardiography (TEE) is a dynamic imaging modality that enables real-time visualization of cardiac structures and function, which is particularly valuable in assessing left ventricular performance, valvular status, and volume status during surgery (3). Previous studies have demonstrated that TEE significantly enhances intraoperative decision-making accuracy in cardiac procedures (4). The FloTrac system, a minimally invasive cardiac output monitoring technology based on arterial pulse contour analysis, calculations of cardiac output (CO) and cardiac index (CI) using arterial pressure waveforms, allows for continuous and real-time hemodynamic assessment (5). While FloTrac has shown favorable monitoring performance in non-cardiac surgeries (6), its utility in high-risk cardiac surgeries requires further investigation. The combined use of TEE and FloTrac during cardiac valve replacement offers a complementary approach, integrating dynamic cardiac imaging with continuous hemodynamic monitoring.

To date, studies have explored the individual application of either TEE or FloTrac in cardiac surgery. TEE has been shown to effectively evaluate left ventricular function and guide volume management (7), while FloTrac has demonstrated reliable hemodynamic prediction in non-cardiac surgical settings (8). However, limited research has addressed the combined use of TEE and FloTrac specifically in cardiac valve replacement, and evidence from retrospective matched-cohort cohort studies remains scarce.

The present study compared TEE combined with FloTrac to TEE with invasive arterial blood pressure monitoring in cardiac valve replacement surgery. The objective is to clarify the advantages and clinical value of the TEE + FloTrac approach in this complex surgical context.

## 2 Materials and methods

### 2.1 Study objects

This was a retrospective matched cohort study. Electronic medical records from January 2021 to June 2025 were reviewed. During this period, 81 patients who underwent TEE combined with FloTrac monitoring were identified and included as the TEE + FloTrac group. Using 1:1 individual matching, another 81 patients who received TEE combined with invasive arterial blood pressure monitoring during the same period were selected as the TEE + Invasive Arterial Pressure group. Inclusion criteria were as follows: (1) patients meeting the diagnostic criteria for valvular heart disease according to relevant clinical guidelines (9) and scheduled for valve replacement surgery; (2) patients assessed by cardiothoracic surgeons to be eligible for cardiopulmonary bypass-assisted valve replacement; and (3) patients with complete postoperative follow-up data available in the medical records for at least 30 days; and (4) with American Society of Anesthesiologists (ASA) physical status classification III–IV.

Exclusion criteria included: (1) patients with severe comorbidities such as end-stage liver disease, renal failure, severe pulmonary disease, or multiple organ dysfunction syndrome who were unable to tolerate surgery; (2) patients with severe left ventricular dysfunction, e.g., left ventricular ejection fraction (LVEF) <30% as measured by transthoracic echocardiography within 30 days prior to surgery due to their significantly higher perioperative risk profile and potential impact on hemodynamic parameter interpretation; patients undergoing emergency surgery for conditions such as acute myocardial infarction (<30 days before surgery) or acute aortic dissection; patients with uncontrolled arrhythmias posing significant hemodynamic instability (e.g., rapid ventricular response atrial fibrillation refractory to medical management); (3) patients with severe postoperative complications (e.g., major infection, postoperative stroke) affecting pain or cognitive assessment; and (4) patients with specific technical contraindications to monitoring, such as failed radial artery cannulation or contraindications to TEE probe placement (e.g., esophageal stricture, recent upper GI surgery). This study was approved by the hospital's ethics committee and conducted in accordance with the ethical principles of the Declaration of Helsinki. The requirement for informed consent was waived.

Although retrospective in design, a *post hoc* sample size calculation was performed for the primary endpoint of postoperative cognitive dysfunction (POCD) within 30 days. The sample size calculation was based on a previous report (10). Assuming a POCD incidence of 18.5% in the conventional monitoring group vs. 9.9% in the combined monitoring group, with *α* = 0.05 and *β* = 0.20, the sample size of 81 patients per group would be able to detect an absolute risk reduction of approximately 8.6% (corresponding to an odds ratio of 0.48) under the specified conditions.

**Figure 1:**
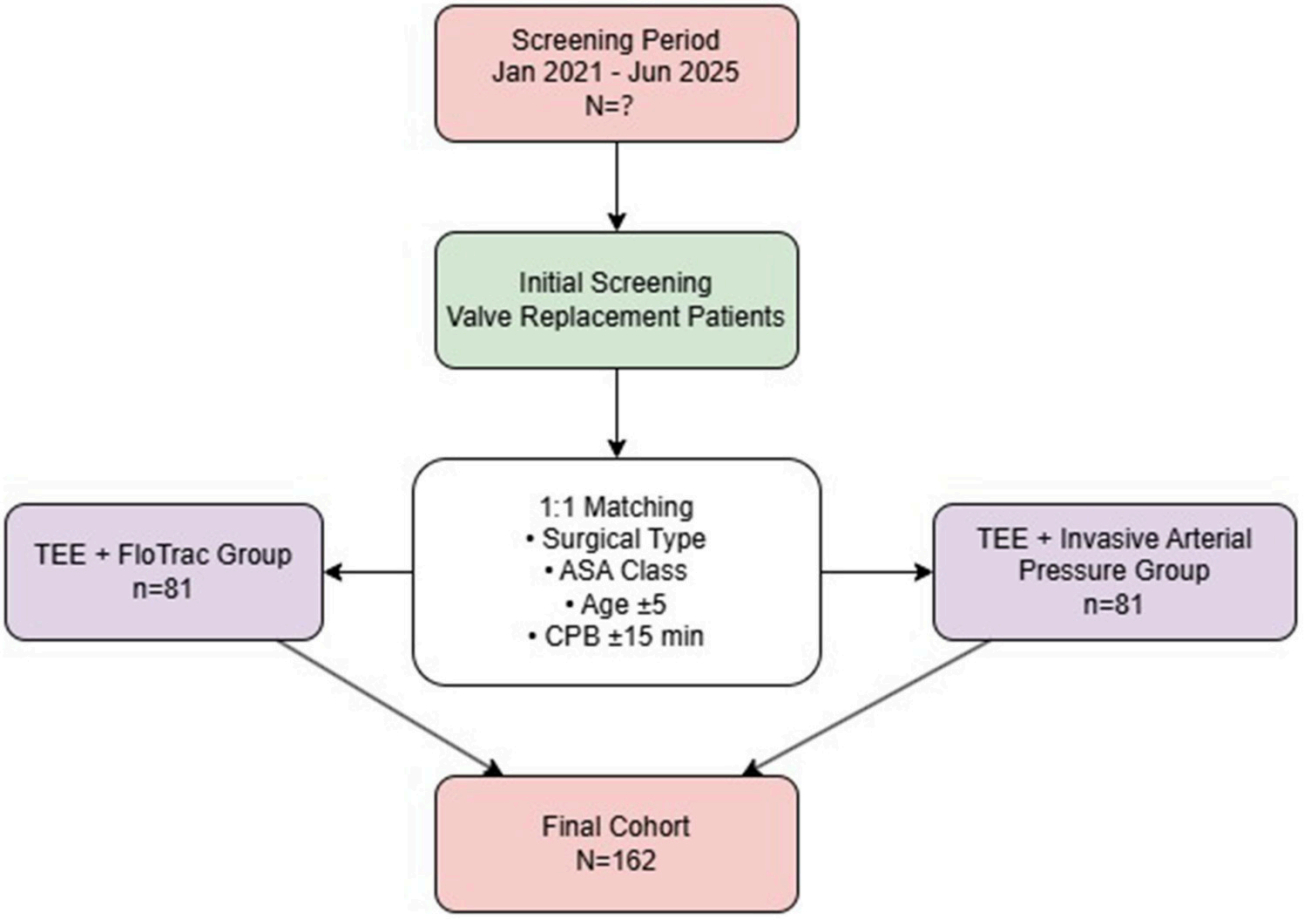
Patient selection and matching flowchart for the retrospective matched cohort study.

Ultimately, 81 patients per group (total *n* = 162) were included in the analysis. Patients in the control group were matched based on surgical type and ASA classification (exact matching), as well as age (±5 years) and cardiopulmonary bypass time (±15 min) (tolerance matching). Standardized mean difference (SMD) was used to assess balance after matching, with all SMD values <0.1 indicating good balance. The patient selection and matching process is illustrated in Figure 1.

### 2.2 Anesthesia protocol

Upon entering the operating room, all patients received standard monitoring, including electrocardiogram, heart rate (HR), invasive arterial pressure, and peripheral oxygen saturation. Anesthesia induction included intravenous administration of: 0.03 mg/kg Midazolam, 0.3 mg/kg Etomidate, 1 µg/kg Sufentanil, and 0.15 mg/kg Cisatracurium besylate. After endotracheal intubation, mechanical ventilation was initiated using intermittent positive pressure ventilation, with tidal volume set at 8 ml/kg, respiratory rate (RR) of 10–14 breaths/min, end-tidal CO_2_ (PETCO_2_) maintained at 35–40 mmHg, and inspired oxygen concentration at 50%.

Anesthesia maintenance was achieved with: Sevoflurane inhalation (1%–2%), intravenous infusion of cisatracurium besylate at 0.2 mg/kg/h, Dexmedetomidine at 1 µg/kg/h, with additional sufentanil (1–2 µg/kg) as needed. The bispectral index (BIS) was maintained between 40 and 60 throughout the procedure (11).

### 2.3 Hemodynamic monitoring

A three-lumen central venous catheter was inserted under ultrasound guidance into the right internal jugular vein in both groups, connected to a CVP transducer. Arterial pressure transducers were connected to the FloTrac system (Edwards Lifesciences, Irvine, CA, USA).

In the TEE + FloTrac group, TEE monitoring was performed using the Philips IE33 ultrasound system (Philips Healthcare, Amsterdam, Netherlands) and compatible TEE probe. Standard imaging planes included the mid-esophageal four-chamber view, transgastric mid–short-axis view, and transgastric long-axis view. Left ventricular dimensions were measured at end-systole and end-diastole from M-mode images. The difference multiplied by HR yielded cardiac output (CO), which was normalized to body surface area to obtain cardiac index (CI). Pulse Doppler was used in the transgastric long-axis view to measure blood flow velocity across the left ventricular outflow tract (LVOT). The velocity-time integral (VTI) was calculated from the spectral tracing. LVOT diameter (D) was measured in the mid-esophageal long-axis view, and cross-sectional area was computed as: LVOT = π (D/2)^2^. Stroke volume (SV) was then derived as: SV = VTI × LVOT. All TEE procedures were performed by the same professionally trained operator. FloTrac monitoring provided continuous real-time measurements of CO, CI, and systemic vascular resistance index (SVRI) based on arterial waveform analysis.

In the TEE + Invasive Arterial Pressure group, invasive blood pressure monitoring was performed using the Philips IntelliVue MP70 monitor (Philips Healthcare, Amsterdam, Netherlands). A radial arterial catheter was placed to monitor invasive arterial pressure (AP), CVP, HR, and other vital signs. TEE monitoring was conducted identically to the TEE + FloTrac group.

### 2.4 Data collection and monitoring indicators

Baseline data included patient age, weight, LVEF, and comorbidities (e.g., diabetes mellitus, hypertension, congestive heart failure). Intraoperative data included operative time, cardiopulmonary bypass (CPB) duration, aortic cross-clamp time, time from cardiac resuscitation to CPB termination, and type and dosage of vasoactive medications.

Hemodynamic parameters were recorded at four key time points: T1: After anesthesia induction and monitoring line placement, before surgical incision; T2: 10 min after initiation of partial CPB flow (partial flow reestablishment); T3: At the termination of CPB, immediately prior to weaning and decannulation; T4: 6 h postoperatively. Parameters recorded at each time point included: Mean Arterial Pressure (MAP), Central Venous Pressure (CVP), HR, SV, CO, CI, SVRI, Stroke Volume Variation (SVV), Velocity-Time Integral (VTI), Left Ventricular End-Diastolic Volume (LVEDV), and Fraction of Area Change (FAC). At T4 (6 h postoperatively), TEE was not routinely performed; therefore, VTI, FAC, and LVEDV were not available. Only FloTrac-derived parameters (SV, SVV, CO, CI, SVRI) and standard monitoring indices (MAP, CVP, HR) were recorded.

Within 30 days postoperatively, outcomes recorded included POCD and major complications [low cardiac output syndrome, new-onset atrial fibrillation, acute kidney injury [AKI], and stroke or transient ischemic attack [TIA]]. AKI was defined according to the KDIGO criteria: an increase in serum creatinine by ≥0.3 mg/dl within 48 h, or an increase to ≥1.5 times the baseline value, or urine output <0.5 ml/kg/h for at least 6 consecutive hours.

Low cardiac output syndrome was defined as a cardiac index <2.0 L/min/m^2^ requiring inotropic support or mechanical circulatory assistance.

New-onset atrial fibrillation was defined as a postoperative episode of AF lasting ≥30 s, documented by continuous electrocardiographic monitoring, in patients without a prior history of AF.

POCD was identified retrospectively from medical records based on a documented diagnosis by neurologists or psychiatrists, supported by standardized neurocognitive assessments (MMSE or MoCA) performed during routine postoperative follow-up. Baseline cognitive assessment was conducted within 3 days before surgery, and postoperative assessment was performed within 7 days (±2 days). POCD was defined as a decline of ≥2 points in MMSE or ≥3 points in MoCA compared with baseline, consistent with published thresholds for clinically significant cognitive decline.

The primary analysis focused on POCD incidence within 30 days. Secondary analyses included differences in intraoperative hemodynamic parameters (SV, CO, CI, SVRI) across T1–T4, and postoperative complications (POCD, low CO syndrome, new-onset AF, AKI, stroke/TIA), and an exploratory analysis assessing the potential of hemodynamic parameters as predictors of POCD. As the study was not preregistered and no predefined primary endpoint had been specified in advance, these endpoints should be regarded as *post hoc* definitions, and all analyses are considered exploratory and hypothesis-generating.

### 2.5 Statistical analysis

All statistical analyses were performed using SPSS version 26.0 (IBM Corp., Armonk, NY, USA) and Python version 3.11.6 (Python Software Foundation, Wilmington, DE, USA), with key packages including pandas 2.1.1, numpy 1.26.0, and statsmodels 0.14.0 for data processing and regression analyses. Continuous variables were expressed as mean ± SD or median [M (P25, P75)]. For longitudinal hemodynamic data, linear mixed-effects models (LMM) were employed. To account for the matched-pair design, models were initially fitted with a random intercept for matched pairs; however, the estimated variance for pairs was negligible, thus the final models included only a subject-specific random intercept to handle repeated measures. Fixed effects included group, time, and their interaction. A pre-specified analysis plan focused on CI and SV, including their group main effects, group × time interactions, and inter-group differences at T1–T3. The False Discovery Rate (FDR) procedure was applied to this family of tests, with corrected *p*-values (*q*-values) reported for these primary contrasts. All other model outputs and the T3 correlation analysis are considered exploratory and are presented without multiplicity adjustment. For the primary endpoint POCD, conditional logistic regression respecting the matched pairs was used for group comparisons. To explore factors associated with POCD, an exploratory ordinary logistic regression was employed. This exploratory model did not preserve the matched design due to the inclusion of multiple continuous hemodynamic covariates and the limited number of events. A sensitivity analysis using conditional logistic regression (preserving matching) confirmed the robustness of the group effect estimate (see Supplementary material). A two-sided *p* (or *q*) < 0.05 was considered statistically significant.

## 3 Results

### 3.1 Comparison of baseline characteristics

A total of 162 patients were included, with 81 patients in the TEE + FloTrac group and 81 in the TEE + invasive arterial pressure monitoring group. Control group patients were selected via 1:1 individual matching: exact matching for surgical type and ASA physical status, and tolerance matching for age (±5 years) and CPB time (±15 min).

Standardized mean differences (SMDs) for all preoperative variables ranged from 0.04 to 0.09, with no value exceeding 0.1, confirming good balance between groups (Table 1). No statistically significant differences were observed for age (SMD = 0.08, *p* = 0.56), sex (SMD = 0.04, *p* = 0.748), BMI (SMD = 0.07, *p* = 0.248), LVEF (SMD = 0.04, *p* = 0.777), ASA classification (SMD = 0.05, *p* = 0.703), predicted CPB time (SMD = 0.09, *p* = 0.598), diabetes (SMD = 0.08, *p* = 0.603), or hypertension (SMD = 0.06, *p* = 0.637). Intraoperative indicators, including surgical duration, actual CPB time, aortic cross-clamp time, and vasoactive drug use, were analyzed separately (Table 2). Overall, baseline characteristics appeared comparable between the two groups.

**Table 1:** Baseline characteristics comparison (preoperative variables only).

Variables	TEE + FloTrac group (*n* = 81)	TEE + invasive arterial pressure group (*n* = 81)	*t/χ* ^2^	*P*-value	SMD
Age (years)	60.07 ± 12.23	65.56 ± 10.86	−0.585	0.560	0.08
Sex (Male/Female)	48/33	50/31	0.103	0.748	0.04
BMI (kg/m^2^)	25.00 ± 3.13	24.45 ± 2.89	1.159	0.248	0.18
LVEF (%)	58.26 ± 6.33	58.00 ± 5.14	0.283	0.777	0.04
Diabetes mellitus [*n* (%)]	25 (30.9%)	22 (27.2%)	0.27	0.603	0.08
Hypertension [*n* (%)]	40 (49.4%)	43 (53.1%)	0.222	0.637	0.07
ASA classification [*n* (%)]			0.145	0.703	0.05
III	52 (64.2%)	50 (61.7%)			
IV	29 (35.8%)	31 (38.3%)			
Predicted CPB time (min)^a^	180.52 ± 52.31	185.17 ± 48.69	−0.528	0.598	0.09

^a^ (1) Predicted CPB time refers to the estimated value used for tolerance matching during group assignment; (2) All SMD values < 0.1, confirming balanced baseline characteristics after matching; (3) ASA classification was included as a key exact-matching variable to explicitly verify the effectiveness of matching.

**Table 2:** Intraoperative indicators comparison.

Variables	TEE + FloTrac group (*n* = 81)	TEE + invasive arterial pressure group (*n* = 81)	*t/χ* ^2^	*P*-value
Surgical duration (min)	345.48 ± 91.30	299.01 ± 58.77	1.044	0.298
Actual CPB time (min)	182.31 ± 54.72	197.13 ± 50.43	−1.311	0.192
Aortic cross-clamp time (min)	142.48 ± 52.05	130.04 ± 49.21	1.167	0.245
Use of vasoactive drugs [*n* (%)]	28 (34.6%)	30 (37.0%)	0.107	0.743

Actual CPB time refers to the recorded duration during surgery, distinguished from the predicted CPB time used for matching.

### 3.2 Comparison of hemodynamic parameter changes between groups

As detailed in the Methods, the reported linear mixed-effects model results are based on models incorporating a subject random intercept, which were determined to be the most appropriate after evaluation of the matched-pair design showed no substantial pair-level correlation.

Linear mixed-effects models were fitted to analyze the hemodynamic trajectories. In line with our pre-specified analysis plan, the primary focus was on CI and SV, with FDR correction applied to the corresponding tests. The complete, unadjusted results for all parameters are available in Supplementary Table S1.

A significant group × time interaction was observed for the pre-specified primary parameter, Cardiac Index (*F* = 18.10, *q* < 0.001), indicating distinct temporal patterns between the two monitoring strategies. The group main effect for CI was also significant (*F* = 16.49, *q* < 0.001). For the pre-specified secondary parameter, Stroke Volume, the group × time interaction showed a non-significant trend after FDR correction (*F* = 5.26, *q* = 0.054). The detailed results for all pre-specified contrasts, including inter-group differences at individual time points, are provided in Supplementary Table S2. Among other parameters not part of the pre-specified primary testing, significant group effects were found for HR and SVV after FDR correction (*q* = 0.024 and *q* < 0.001, respectively). The summarized results of the key pre-specified contrasts are presented in Table 3.

**Table 3:** Primary analysis of key hemodynamic parameters using linear mixed-effects models (FDR-corrected).

Parameter	Effect type	*F*-value	Original *p*-value	FDR-corrected q-value	Significance
CI	Group × Time	18.1	<0.001	<0.001	**
	Group Main Effect	16.49	<0.001	<0.001	**
SV	Group × Time	5.26	0.001	0.054	ns
	Group Main Effect	1.01	0.316	0.421	ns
HR	Group Main Effect	6.79	0.009	0.024	*
SVV	Group Main Effect	18.73	<0.001	<0.001	**

* Indicates *q* < 0.05.

** Indicates *q* < 0.01.

*** Indicates *q* < 0.001, ns indicates not significant.

This table presents results for pre-specified primary (CI) and secondary (SV) parameters, along with other significant findings after FDR correction.

*q* < 0.05 is considered significant.

FDR, false discovery rate.

### 3.3 Pearson correlation analysis

**Figure 2:**
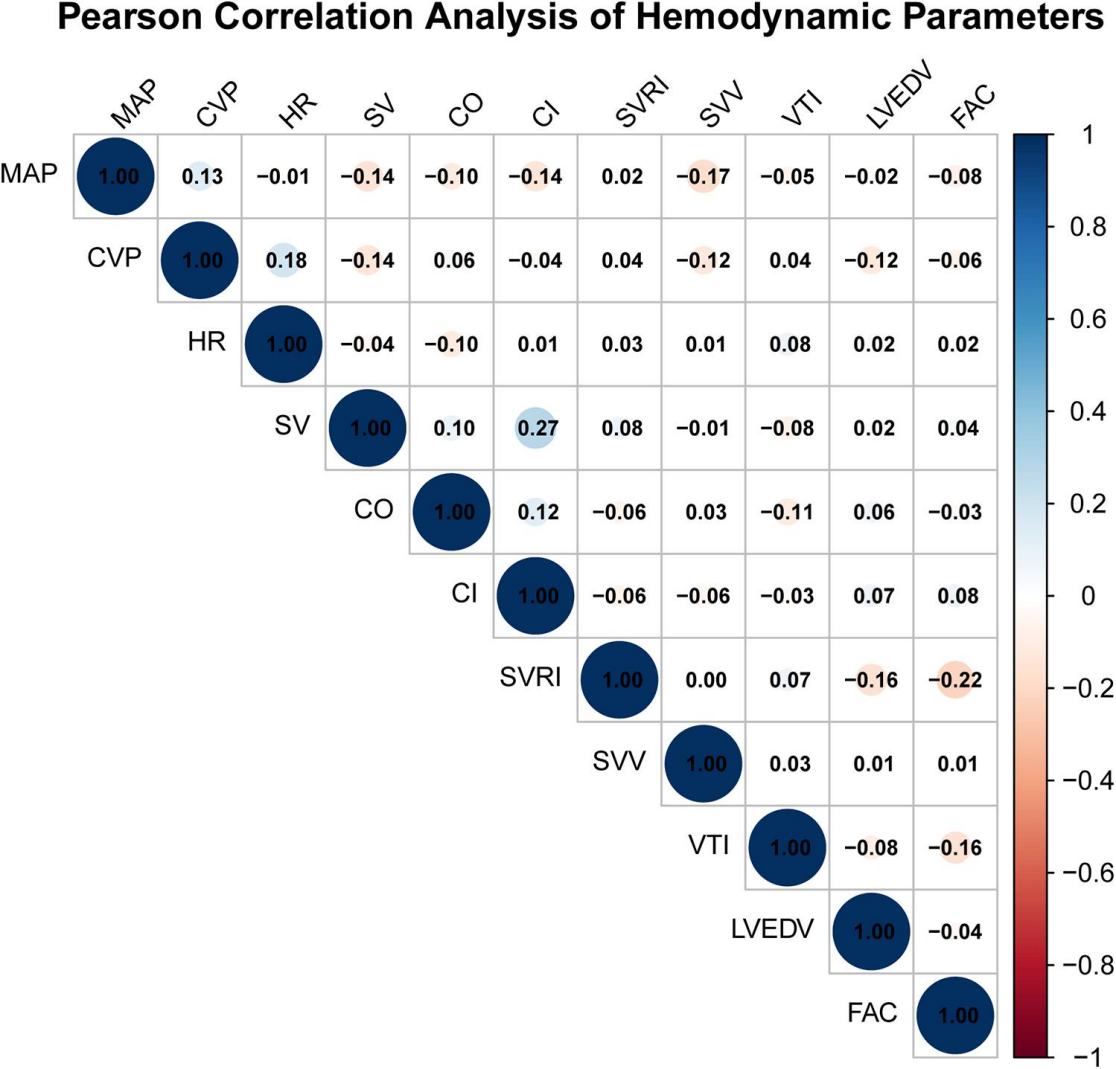
Pearson correlation analysis of hemodynamic parameters.

Pearson correlation analysis was performed at T3 (post-CPB) to explore potential linear associations between selected hemodynamic parameters derived from TEE and FloTrac monitoring. Correlation coefficients (r) with 95% confidence intervals (CIs) were calculated for all parameter pairs. Most correlations were weak and not statistically significant (|*r*| < 0.2, *p* > 0.05). Two exceptions were observed: SV and CI (*r* = 0.274, 95% CI 0.123–0.412, *p* = 0.0004) and FAC and SVRI (*r* = −0.220, 95% CI −0.360–0.075, *p* = 0.0049). These results indicate that although a few parameter pairs showed statistically significant correlations, the overall strength of linear associations was weak. No adjustment for multiple comparisons was applied due to the exploratory nature of the analysis. We emphasize that correlation does not imply clinical agreement, interchangeability, or causal relationship between the two monitoring methods. These findings solely describe statistical associations and should not be interpreted as evidence of measurement equivalence (Figure 2).

### 3.4 Comparison of postoperative complications and adverse outcomes

In the matched cohort of 81 pairs, no statistically significant differences were observed between the TEE + FloTrac group and the TEE + invasive arterial pressure group in postoperative complications or adverse outcomes. The incidence of POCD was 9.9% vs. 18.5% (paired OR = 0.48, 95% CI: 0.19–1.21; McNemar *P* = 0.115), low cardiac output syndrome 7.4% vs. 14.8% (OR = 0.46, 95% CI: 0.16–1.29; *P* = 0.134), new-onset atrial fibrillation 27.2% vs. 30.9% (OR = 0.84, 95% CI: 0.42–1.65; *P* = 0.603), AKI 9.9% vs. 6.2% (OR = 1.67, 95% CI: 0.52–5.33; *P* = 0.386), and stroke/TIA 2.5% vs. 5.0% (OR = 0.49, 95% CI: 0.09–2.74; *P* = 0.405). McNemar's test accounted for the paired design. Conditional logistic regression produced similar effect directions without statistical significance (Table 4). A sensitivity analysis using conditional logistic regression yielded an identical OR and CI (see Supplementary Table S3), confirming the robustness of this null finding.

**Table 4:** Comparison of postoperative complications and adverse outcomes between the two groups (*n*, %).

Complications	TEE + FloTrac group (*n* = 81)	TEE + Invasive Arterial Pressure group (*n* = 81)	Discordant pairs (b/c)	Paired OR (95% CI)	McNemar's *P* value
POCD	8 (9.9%)	15 (18.5)	3/10	0.482 (0.192–1.210)^a^	0.115
Low cardiac output syndrome	6 (7.4%)	12 (14.8%)	2/8	0.460 (0.164–1.292)^a^	0.134
New-onset atrial fibrillation	22 (27.2%)	25 (30.9%)	6/9	0.835 (0.423–1.648)^a^	0.603
AKI	8 (9.9%)	5 (6.2%)	5/2	1.666 (0.521–5.327)^a^	0.386
Stroke/TIA	2 (2.5%)	4 (5.0%)	1/3	0.487 (0.087–2.738)^a^	0.405

^a^ Paired OR and 95% CI estimated from conditional logistic regression; McNemar's test used for unadjusted paired binary comparison.

### 3.5 Univariate and multivariate logistic regression analysis of postoperative POCD

To explore factors associated with POCD, we performed an exploratory analysis using univariate and multivariate ordinary logistic regression (Tables 5, 6). This approach was chosen due to the limited number of POCD events and the inclusion of multiple continuous hemodynamic covariates, which can lead to model instability in conditional logistic regression. Given the exploratory, hypothesis-generating nature of this analysis and the fact that the matching variables were well-balanced between groups (Tables 1, 2), ordinary logistic regression was deemed appropriate for assessing potential associations.

**Table 5:** Univariate logistic regression analysis of factors associated with POCD.

Variable	B	SE	OR	95% CI	*P*
Surgery_Time	−0.006	0.003	0.994	0.988–0.999	0.045
CI_T3	0.935	0.575	2.548	0.826–7.861	0.104
type	−0.729	0.47	0.482	0.192–1.210	0.120
VTI_T3	0.200	0.137	1.221	0.933–1.598	0.146
SVRI_T2	−0.002	0.002	0.998	0.995–1.001	0.181
CO_T3	−0.406	0.337	0.666	0.344–1.290	0.228
Age	−0.039	0.035	0.962	0.897–1.030	0.266
LVEDV_T3	0.018	0.018	1.018	0.983–1.055	0.322
HR_T3	0.012	0.013	1.013	0.987–1.039	0.335
SVV_T1	−0.08	0.093	0.923	0.770–1.108	0.391
MAP_T2	0.019	0.027	1.02	0.967–1.075	0.471
CO_T2	0.214	0.33	1.238	0.648–2.365	0.517
Aortic_Clamp_Time	0.003	0.005	1.003	0.994–1.012	0.518
SVRI_T3	−0.001	0.002	0.999	0.996–1.002	0.526
CPB_Time	0.003	0.004	1.003	0.994–1.011	0.532
CVP_T2	0.146	0.245	1.157	0.716–1.870	0.551
Baseline_LVEF	0.018	0.04	1.018	0.942–1.100	0.647
FAC_T3	−0.021	0.054	0.98	0.880–1.090	0.705
SV_T3	−0.007	0.023	0.993	0.949–1.039	0.766
CVP_T3	−0.041	0.15	0.96	0.715–1.288	0.784
MAP_T3	0.004	0.019	1.004	0.968–1.042	0.824
CI_T2	0.129	0.691	1.137	0.294–4.406	0.852
BMI	−0.012	0.075	0.988	0.853–1.144	0.87
SV_T2	0.004	0.025	1.004	0.956–1.054	0.877

**Table 6:** Multivariate logistic regression analysis of factors associated with POCD.

Constant	B	SE	OR	95% CI	*P*
Surgery_Time	−0.007	0.003	0.993	0.987–0.999	0.032
CI_T3	1.083	0.593	2.953	0.923–9.447	0.068
Cconst	−2.274	1.666	0.103	0.004–2.692	0.172

This analysis avoids stepwise selection, prevents overfitting by limiting variables according to the events-per-variable (EPV) principle, and does not include post-treatment variables without clear clinical relevance for early prediction.

To explore factors associated with POCD, we first performed univariate logistic regression analysis including baseline variables (Age, BMI, Baseline_LVEF, Surgery_Time, CPB_Time, Aortic_Clamp_Time, and group) and intraoperative hemodynamic parameters (MAP, CVP, HR, SV, CO, CI, SVRI, SVV, VTI, LVEDV, FAC) measured at clinically relevant time points (T1–T3). The study group variable indicated whether the patient belonged to the TEE + FloTrac group or the TEE + Invasive Arterial Pressure group. Variables with the smallest *P* values in univariate analysis were selected for multivariate modeling, taking into account the limited number of events (*n* = 23) to avoid overfitting (Table 5).

In univariate analysis, Surgery_Time was significantly associated with POCD (OR = 0.994, 95% CI: 0.988–0.999, *P* = 0.045). Other variables, including baseline characteristics and hemodynamic parameters at T1–T3, did not reach statistical significance (*P* > 0.05), although some showed trends (e.g., CI_T3 OR = 2.548, 95% CI: 0.826–7.861, *P* = 0.104).

A parsimonious multivariate model including Surgery_Time and CI_T3 was then constructed. In this model, Surgery_Time remained statistically significant (OR = 0.993, 95% CI: 0.987–0.999, *P* = 0.032), while CI_T3 showed a trend toward higher risk of POCD without reaching statistical significance (OR = 2.953, 95% CI: 0.923–9.447, *P* = 0.068) (Table 6).

These results suggest that longer surgery time is independently associated with a lower likelihood of postoperative POCD, whereas intraoperative cardiac index at T3 may have a potential but non-significant effect. No predictive claims are made due to limited events, and post-T3 measurements (including T4) were not considered in the model to respect the “early prediction” landmark. All analyses were conducted respecting the matched study design.

## 4 Discussion

Accurate hemodynamic management during cardiac valve replacement surgery is important for intraoperative safety and postoperative recovery. Conventional monitoring methods, such as invasive arterial blood pressure measurement, provide basic circulatory information but are limited in assessing cardiac function and volume status changes (12). TEE allows real-time evaluation of cardiac structure and function, offering valuable intraoperative guidance. The FloTrac system, which derives continuous estimates of CO, SV, and other parameters based on arterial waveform analysis, facilitates dynamic adjustments of fluid therapy and vasoactive medications (13). This study examined the combined use of TEE and FloTrac during valve replacement surgery, focusing on hemodynamic monitoring, postoperative complications, and exploratory risk assessment of POCD.

Mixed-effects analysis revealed significant group, time, and interaction effects across multiple hemodynamic parameters. Notably, differences in SV, CO, and CI between the two monitoring strategies suggest that FloTrac may be more sensitive for detecting changes in stroke volume and cardiac output during early perfusion and weaning phases, while TEE provides complementary insights into ventricular volumes and wall motion. These findings are consistent with prior studies indicating that TEE enables detailed assessment of cardiac structure and function, whereas FloTrac offers continuous monitoring of dynamic trends (14–16). The combination of these modalities therefore provides complementary hemodynamic perspectives, which may support individualized intraoperative decision-making.

Pearson correlation analysis at T3 demonstrated only weak associations between corresponding TEE- and FloTrac-derived parameters. These correlations were interpreted as exploratory associations only, without implying agreement or interchangeability between the two modalities. The observed weak correlations likely reflect differences in measurement principles: FloTrac relies on arterial waveform analysis and algorithmic estimations sensitive to vascular compliance and arterial tone (17), whereas TEE-derived indices depend on geometric assumptions and operator technique (18). These results suggest that data from the two modalities provide complementary perspectives, but should not be considered equivalent or interchangeable.

From a technical standpoint, FloTrac appeared more suited for tracking dynamic trends (e.g., CO, SVV, SV), while TEE was more appropriate for anatomical assessments (e.g., LVEDV, FAC, VTI) and regional ventricular function (19). In clinical practice, parameters such as CVP and CO may track trends in parallel across modalities, but they are not interchangeable. However, for parameters like SVRI and LVEDV, a consistent monitoring approach is recommended, supplemented by clinical judgment to avoid misinterpretation due to differences in measurement principles.

With respect to postoperative complications, we observed no statistically significant differences between groups in the incidence of POCD, low cardiac output syndrome, atrial fibrillation, AKI, or cerebrovascular events. The observed rate of POCD (overall 14.2%) falls within the wide range reported in previous literature (5%–50%) (20, 21). Exploratory logistic regression analysis suggested that longer Surgery_Time was associated with lower likelihood of POCD, while higher CI at T3 showed a non-significant trend toward increased risk; other baseline and intraoperative hemodynamic parameters at T1–T3 were not significantly associated with POCD. These results indicate that intraoperative hemodynamic parameters alone, measured at isolated time points, are insufficient to explain postoperative cognitive decline. Prior studies have emphasized that POCD is multifactorial, involving perioperative inflammation, cerebral oxygen desaturation, and patient-specific susceptibility (22, 23). Our findings therefore reinforce the notion that multimodal risk assessment, integrating hemodynamic monitoring with biomarkers and cerebral oximetry, may be required to understand and manage POCD.

In summary, TEE and FloTrac each offer unique advantages: TEE provides high-resolution visualization of ventricular function and valvular dynamics, while FloTrac delivers continuous trend monitoring of cardiac output and related indices. Their combined use provides a more comprehensive dataset for intraoperative hemodynamic management. However, the exploratory analysis of POCD highlights the current limitations of hemodynamic parameters as sole predictors, and underscores the need for larger, preregistered retrospective matched-cohort studies incorporating multimodal data to validate these preliminary observations.

## 5 Limitation

This study has several limitations. First, its retrospective, matched-cohort design at a single center limits the generalizability of the findings. Although matching was employed to enhance comparability, residual and unmeasured confounding factors (e.g., subtle differences in surgical technique or anesthesia management) may persist, and any postoperative exclusions could introduce selection bias. The modest sample size and low incidence of postoperative complications, particularly POCD, limited the statistical power to detect differences in clinical outcomes between the monitoring strategies. In addition, the study was not preregistered, and no predefined primary endpoint was established. Therefore, all analyses, including the exploratory modeling of POCD, should be regarded as hypothesis-generating.

Second, the subjective nature of TEE-based measurements (e.g., FAC, LVEDV) introduces potential operator-dependent variability, despite all examinations being performed by an experienced echocardiographer.

Third, data availability varied across time points. At T4 (6 h postoperatively), TEE was not routinely performed, so parameters such as VTI, LVEDV, and FAC were not measured for most patients and were excluded from the T4 analysis. The available case numbers for each parameter at each time point are reported in the Results section for clarity.

Finally, it is important to reiterate that while some hemodynamic parameters (e.g., CVP, CO) may exhibit parallel trends when measured by TEE and FloTrac, the two methods are based on fundamentally different principles and cannot be considered interchangeable.

## 6 Conclusion

The combined use of TEE and FloTrac provides complementary information for intraoperative hemodynamic monitoring during cardiac valve replacement surgery, allowing more detailed assessment of cardiac function and trends in stroke volume, cardiac output, and systemic vascular resistance. However, these findings represent observational associations rather than causal effects, and the two methods cannot be considered interchangeable based on the current data. This study did not find evidence that one monitoring strategy was superior to the other in reducing postoperative complications, including POCD.

Exploratory analyses of POCD suggested nominal associations of certain hemodynamic parameters with postoperative cognitive outcomes, but predictive performance was limited and effect sizes were unstable. Therefore, these findings should be interpreted as hypothesis-generating rather than confirmatory.

Future studies should focus on retrospective matched-cohort, preregistered trials with standardized monitoring protocols, larger sample sizes, and multimodal data integration—including hemodynamic, cerebral oximetry, and biomarker measurements—to more accurately evaluate perioperative management strategies and postoperative cognitive risk.

## Data Availability

The original contributions presented in the study are included in the article/Supplementary Material, further inquiries can be directed to the corresponding author.
