# Supplementary material for: Application of transesophageal echocardiography combined with FloTrac monitoring in cardiac valve replacement surgery
Source: Front Cardiovasc Med. 2025 Oct 31;12:1667017. doi: 10.3389/fcvm.2025.1667017 (PMC12615405; doi:10.3389/fcvm.2025.1667017)
Supplement: Supplementary file 1 [file Table1.docx]

Supplementary Table S1. Complete Linear Mixed-Effects Model Results for All Hemodynamic Parameters (Unadjusted)

| **Parameters** | **Time points** | **TEE + FloTrac group (n=81)** | **TEE +Invasive Arterial Pressure group (n=81)** | ***Group Effect F (p-value)*** | ***Time Effect F (p-value)*** | ***Group × Time Interaction F (p-value)*** |
| --- | --- | --- | --- | --- | --- | --- |
| **MAP (mmHg)** | T1 | 86.52±11.59 | 84.22±10.53 | 3.88 (0.051) | 2.64 (0.049)* | 4.48 (0.004)* |
|  | T2 | 81.58±8.58 | 84.7±8.14 |  |  |  |
|  | T3 | 84.54±12.37 | 80.34±11.11 |  |  |  |
|  | T4 | 84.86±10.44 | 81.13±10.32 |  |  |  |
| **CVP (mmHg)** | T1 | 8.09±1.16 | 8.30±1.32 | 0.09 (0.768) | 2.38 (0.069) | 5.23 (0.001)* |
|  | T2 | 8.59±0.88 | 8.24±0.95 |  |  |  |
|  | T3 | 8.82±1.52 | 8.32±1.47 |  |  |  |
|  | T4 | 8.22±1.57 | 8.73±1.57 |  |  |  |
| **HR (beats/min)** | T1 | 73.4±8.61 | 71.3±8.71 | 6.79 (0.009)* | 303.50 (<0.001)* | 3.53 (0.015)* |
|  | T2 | 71.68±9.03 | 73.28±8.03 |  |  |  |
|  | T3 | 94.39± 15.71 | 99.69±17.94 |  |  |  |
|  | T4 | 98.99± 8.46 | 103.47±9.61 |  |  |  |
| **SV (mL)** | T1 | 71.66±10.08 | 70.9±11.98 | 1.01 (0.316) | 4.67 (0.003)* | 5.26 (0.001)* |
|  | T2 | 66.66±8.37 | 70.74±9.26 |  |  |  |
|  | T3 | 66.24±9.29 | 69.5±10.21 |  |  |  |
|  | T4 | 69.39±9.7 | 65.92±9.08 |  |  |  |
| **CO (L/min)** | T1 | 4.97±0.59 | 5.01±0.48 | 17.05 (<0.001)* | 15.72 (<0.001)* | 1.39 (0.244) |
|  | T2 | 4.60±0.68 | 4.91±0.66 |  |  |  |
|  | T3 | 4.42±0.6 | 4.66±0.71 |  |  |  |
|  | T4 | 4.54±0.51 | 4.76±0.66 |  |  |  |
| **CI (L/min·m²)** | T1 | 2.54±0.30 | 2.62±0.33 | 16.49 (<0.001)* | 24.15 (<0.001)* | 18.10 (<0.001)* |
|  | T2 | 2.35±0.30 | 2.53±0.33 |  |  |  |
|  | T3 | 2.25±0.31 | 2.59±0.40 |  |  |  |
|  | T4 | 2.36±0.33 | 2.18±0.29 |  |  |  |
| **SVRI (dyn·s·cm⁻⁵·m²)** | T1 | 1460.35±209.46 | 1433.59±182.24 | 2.67 (0.103) | 3.88 (0.009)* | 4.13 (0.007)* |
|  | T2 | 1519.36±148.21 | 1465.24±122.72 |  |  |  |
|  | T3 | 1529.66±168 | 1476.59±126.04 |  |  |  |
|  | T4 | 1459.1±159.37 | 1511.97±125.24 |  |  |  |
| **SVV (%)** | T1 | 13.97±2.39 | 11.85±2.05 | 18.73 (<0.001)* | 2.41 (0.066) | 7.73 (<0.001)* |
|  | T2 | 12.90±3.09 | 12.95±2.24 |  |  |  |
|  | T3 | 12.71±2.59 | 12.71±2.38 |  |  |  |
|  | T4 | 13.98±1.99 | 12.81±2.09 |  |  |  |
| **VTI (cm)** | T1 | 21.07±2.09 | 20.69±2.23 | 2.62 (0.108) | 3.75 (0.024)* | 4.18 (0.016)* |
|  | T2 | 20.32±2.19 | 20.64±1.58 |  |  |  |
|  | T3 | 20.76±1.61 | 19.90±1.74 |  |  |  |
| **LVEDV (mL)** | T1 | 112.41±14.28 | 109.69±14.36 | 2.69 (0.103) | 13.94 (<0.001)* | 4.70 (0.010)* |
|  | T2 | 108.69±13.93 | 111.11±14.24 |  |  |  |
|  | T3 | 100.46±13.97 | 106.92±10.45 |  |  |  |
| **FAC (%)** | T1 | 42.16±4.26 | 41.65±5.21 | 0.16 (0.694) | 2.04 (0.131) | 2.41 (0.091) |
|  | T2 | 42.55±3.88 | 42.12±4.96 |  |  |  |
|  | T3 | 40.63±4.36 | 42.04±3.85 |  |  |  |

Note: Data presented as mean ± SD. This table contains the complete, unadjusted results of all exploratory analyses. *p < 0.05 was considered statistically significant for these unadjusted tests. Results should be interpreted with caution. TEE-specific parameters (VTI, LVEDV, FAC) were not measured at T4.
