# Supplementary material for: Application of transesophageal echocardiography combined with FloTrac monitoring in cardiac valve replacement surgery
Source: Front Cardiovasc Med. 2025 Oct 31;12:1667017. doi: 10.3389/fcvm.2025.1667017 (PMC12615405; doi:10.3389/fcvm.2025.1667017)
Supplement: Supplementary file 2 [file Table2.docx]

Supplementary Table S2. Details of Multiple Comparison Correction for Pre-specified Contrasts

| Pre-specified Contrast | Test Statistic | Original p-value | FDR-corrected q-value | Significance |
| --- | --- | --- | --- | --- |
| CI |  |  |  |  |
| Group × Time Interaction | F = 18.10 | < 0.001 | < 0.001 | ** |
| Group Main Effect | F = 16.49 | < 0.001 | < 0.001 | ** |
| Inter-group difference at T1 | t = -1.76 | 0.081 | 0.108 | ns |
| Inter-group difference at T2 | t = -3.82 | < 0.001 | < 0.001 | ** |
| Inter-group difference at T3 | t = -6.,15 | < 0.001 | < 0.001 | ** |
| SV |  |  |  |  |
| Group × Time Interaction | F = 5.26 | 0.001 | 0.054 | ns (trend) |
| Group Main Effect | F = 1.01 | 0.316 | 0.379 | ns |
| Inter-group difference at T1 | t = 0.43 | 0.665 | 0.665 | ns |
| Inter-group difference at T2 | t = -2.92 | 0.004 | 0.016 | * |
| Inter-group difference at T3 | t = -2.10 | 0.037 | 0.074 | ns |

Note: This table details the False Discovery Rate (FDR) correction applied to the family of pre-specified contrasts for Cardiac Index and Stroke Volume. The family included 10 tests (2 main effects/interactions + 3 inter-group comparisons per parameter). * indicates *q* < 0.05, ** indicates *q* < 0.01, *** indicates *q* < 0.001, ns indicates not significant.
