# Supplementary material for: Application of transesophageal echocardiography combined with FloTrac monitoring in cardiac valve replacement surgery
Source: Front Cardiovasc Med. 2025 Oct 31;12:1667017. doi: 10.3389/fcvm.2025.1667017 (PMC12615405; doi:10.3389/fcvm.2025.1667017)
Supplement: Supplementary file 3 [file Table3.docx]

Supplementary Table S3. Sensitivity Analysis of the Association Between Monitoring Group and Postoperative Cognitive Dysfunction (POCD) Using Conditional Logistic Regression

| Model Type | Odds Ratio (OR) for POCD | 95% Confidence Interval | P-value |
| --- | --- | --- | --- |
| Primary Analysis: Conditional Logistic Regression (Preserves Matching) | 0.48 | 0.19 – 1.21 | 0.115 |
| Exploratory Analysis: Ordinary Logistic Regression (Does Not Preserve Matching) | 0.48 | 0.19 – 1.21 | 0.115 |

Note: This sensitivity analysis demonstrates that the estimated effect of the monitoring strategy on POCD risk is consistent in both direction and magnitude, regardless of the statistical model used. The identical results reinforce the conclusion of no statistically significant difference between groups.
